# Biodegradability Study of Modified Chitosan Films with Cinnamic Acid and Ellagic Acid in Soil

**DOI:** 10.3390/polym16050574

**Published:** 2024-02-20

**Authors:** Maria Swiontek Brzezinska, Ambika H. Shinde, Beata Kaczmarek-Szczepańska, Urszula Jankiewicz, Joanna Urbaniak, Sławomir Boczkowski, Lidia Zasada, Magdalena Ciesielska, Katarzyna Dembińska, Krystyna Pałubicka, Marta Michalska-Sionkowska

**Affiliations:** 1 Department of Environmental Microbiology and Biotechnology, Faculty of Biological and Veterinary Sciences, Nicolaus Copernicus University in Torun, Lwowska 1, 87-100 Toruń, Poland; ambikahshinde@gmail.com (A.H.S.); 311947@stud.umk.pl (J.U.); 305303@stud.umk.pl (S.B.); kdembinska@doktorant.umk.pl (K.D.); 2 Department of Biomaterials and Cosmetics Chemistry, Faculty of Chemistry, Nicolaus Copernicus University in Torun, Gagarina 7, 87-100 Toruń, Poland; kaczmarek@umk.pl (B.K.-S.); 503555@doktorant.umk.pl (L.Z.); 294291@stud.umk.pl (M.C.); 3 Department of Biochemistry and Microbiology, Institute of Biology, Nowoursynowska 159, 02-776 Warsaw, Poland; u_jankiewicz@sggw.pl; 4 Department of Conservation and Restoration of Paper and Leather, Nicolaus Copernicus University, ul. Sienkiewicza 30/32, 87-100 Toruń, Poland; krystynap@doktorant.umk.pl

**Keywords:** chitosan films, biodegradation, *Trichoderma*, cinnamic acid, ellagic acid

## Abstract

Currently, natural polymer materials with bactericidal properties are extremely popular. Unfortunately, although the biopolymer material itself is biodegradable, its enrichment with bactericidal compounds may affect the efficiency of biodegradation by natural soil microflora. Therefore, the primary objective of this study was to evaluate the utility of fungi belonging to the genus *Trichoderma* in facilitating the degradation of chitosan film modified with cinnamic acid and ellagic acid in the soil environment. Only two strains (T.07 and T.14) used chitosan films as a source of carbon and nitrogen. However, their respiratory activity decreased with the addition of tested phenolic acids, especially cinnamic acid. Addition of *Trichoderma* isolates to the soil increased oxygen consumption during the biodegradation process compared with native microorganisms, especially after application of the T.07 and T.14 consortium. Isolates T.07 and T.14 showed high lipolytic (55.78 U/h and 62.21 U/h) and chitinase (43.03 U/h and 41.27 U/h) activities. Chitinase activity after incorporation of the materials into the soil was higher for samples enriched with T.07, T.14 and the consortium. The isolates were classified as *Trichoderma* sp. and *Trichoderma koningii.* Considering the outcomes derived from our findings, it is our contention that the application of *Trichoderma* isolates holds promise for expediting the degradation process of chitosan materials containing bactericidal compounds.

## 1. Introduction

Plastic pollution has a significant impact on terrestrial life [1]. It is likely to pose a major threat to plants and animals, and even humans. Due to their resilience, plastics have strong roots in terrestrial ecosystems as building materials and hygiene products, but most importantly as agricultural packaging [2]. Synthetic materials mulch has been used to increase crop yields worldwide, yet the long-term impact of this material residue on soil health and crop production still remains unclear [3]. Although plenty of efforts are being made to curb the impact of synthetic material pollution in aquatic environments, there is limited information on the remediation of agricultural soils. This necessitates the consideration of newer technologies and better alternatives, like biodegradable polymers [4]. Recently, more emphasis has been given to the use of biopolymers in agriculture for seed coating. These function as stabilizing agents and protect seeds from biological and environmental stresses, enhancing the absorption of nutrients by plants and resulting in the promotion of plant growth and yield improvement [5]. Chitosan is a deacetylated derivative of chitin, a natural polymer derived from the shells of crustaceans [6]. Its biodegradability, non-toxicity and antimicrobial activity make it attractive for a variety of applications in the medical and food industries, as well as in agriculture and aquaculture [7]. Chitosan-based films can be modified with various biocidal substances and phenolic acids [8,9,10] allowing their use in long-term storage of perishable products like food, feed or fertilizers. Polymer degradation is a complex process comprising various physical, chemical and biological factors from the ecosystem. The inherent microbiota is actively involved in this process through the production of hydrolytic enzymes, which are responsible for the degradation process. Among the enzymes produced are chitinases, which, in fungi, play a significant role in the degradation of insect exoskeletons and in the growth of fungal cells [11]. However, an important obstruction to this process is the antimicrobial nature of the film. This renders the native flora insufficient to carry out the process.

Bioaugmentation of resources with efficient microbial formulations may prove to be a solution to the problem. In recent years, a number of formulations have been developed to aid the biodegradation of polymers. It is important to select an organism that is competent in nature, non-pathogenic or antagonistic to crops or plants, and native to the soil ecosystem, when agricultural land is to be subjected to remediation [12]. For the significant degradation of chitosan, the bioaugmentation species must possess chitinolytic enzymes in addition to other hydrolytic enzymes [13]. *Trichoderma* spp. seems to be an ideal choice, as it meets the above criteria and is regarded as a major decomposition and rotting agent of agricultural waste, possessing a wide range of enzymes [14,15]. Previous studies and reports have indicated the role and mechanism of action of *Trichoderma* spp. in the degradation of multiple polymers, such as polycaprolactone, polyethylene terephthalate, polylactide and even chitosan-based films modified with tannic acid, ferulic acid and gallic acid [16,17,18,19,20]. However, polymers with newer and more beneficial modifications are continuously being developed and need to be tested for biodegradation before their application.

The aim of this study was to prepare chitosan films modified using cinnamic acid and ellagic acid. Both are phenolic compounds of plant origin, known for their antimicrobial and anticancer potentials [21,22]. Their modification demonstrates the improved potency of the chitosan films in terms of resilience and acceptance due to its origin [23]. *Trichoderma* were isolated from forest soil and their enzymatic activity and biodegradation of chitosan films were tested. Two isolates with the best biodegradation and enzymatic properties were then selected and used to enrich agricultural soil in order to evaluate the effectiveness of the application.

## 2. Materials and Methods

### 2.1. Isolation and Molecular Identification of Fungal Strains

**Figure 1:**
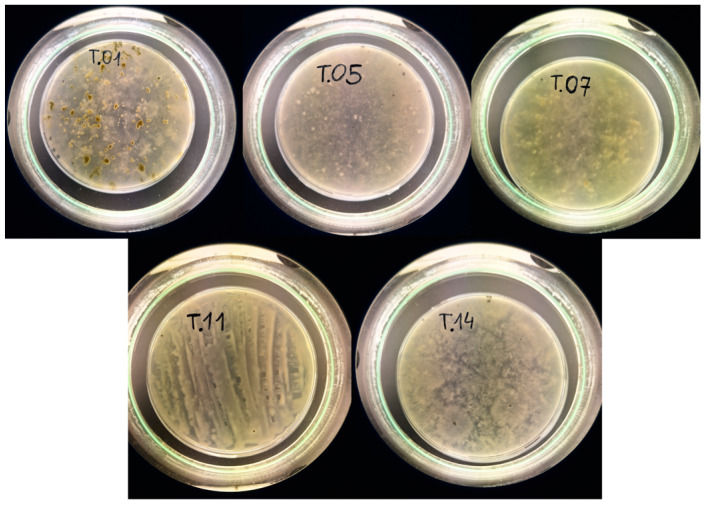
Isolated fungi on PDA medium.

Fungi were isolated from the forest soil of the Kampinoski National Park in Warsaw and were deposited at the Department of Biochemistry and Microbiology, Warsaw University of Life Sciences. Isolates were described as T.01, T.05, T.07, T.11 and T.14. The fungi, stored in microbiological solid potato dextrose agar (PDA) (Difco, Franklin Lakes, NJ, USA) were transferred to a liquid PDA medium and incubated at 25 °C for 5 days (Figure 1). Mycelia fragments were homogenized in liquid nitrogen for molecular identification. The Plant and Fungi DNA Purification Kit (EURx, Gdansk, Poland) was used to perform DNA isolation according to the manufacturer’s written recommendations. The concentration of isolated DNA was measured using a Nanodrop ND-1000 spectrophotometer (Nanodrop Technologies Inc., Wilmington, DE, USA). The amplification reaction primers used for analysis were ITS1 forward (5′ CTTGGTCATTTAGAGGAAGTAA 3′), ITS3 forward (5′ GCATCGATGAAGAACGCAGC 3′) and ITS4 reverse (5′ TCCTCCGCTTATTGATAGC-3′). Sequencing of the polymerase chain reaction products was performed using the Sanger method. The Big Dye Terminator Cycle Sequencing Kit (Applied Biosystems, Thermo Fisher Scientific, Waltham, MA, USA) was used for this purpose. After sequencing, products were precipitated with absolute alcohol (99.8%) (EURx, Poland). The amplified PCR products were sequenced using the Sanger method (Genomed, Warsaw, Poland). The obtained sequences were compared with those contained in NCBI’s GenBank (https://blast.ncbi.nlm.nih.gov/Blast.cgi, accessed on 22 January 2024).

### 2.2. Determination of Enzymatic Activity of Fungi

The most important hydrolytic enzymes involved in the degradation of organic matter were determined as follows: lipase, aminopeptidase, α, β-glucosidase and chitinase [24,25,26]. For this purpose, a fluorimetric method was used, with substrates labelled with MUF (4-methylumbelliferyl) or MCA (7-amino-4-methylcoumarin) molecules, respectively, as follows: MUF-butyrate, MCA-leucine, MUF-α-D-glucoside, MUF-β-D-glucoside and MUF-N-acetyloglucosaminide. The fungi were cultivated on medium containing (g/L) sucrose 10 and yeast extract 3, the medium was inoculated with fungal spores (10^5^/mL) and incubated for 4 days at 25 °C. The culture was then centrifuged (10 min, 10,000× *g*) and the enzyme activity in the supernatant was determined. The reaction mixture contained 2 mL of supernatant and 0.2 mL of the substrate. The final substrate concentration in the sample was 50 µmol/mL. The control was prepared in the same way as the test samples. Prior to substrate addition, the supernatant was inactivated by heating for 10 min at 100 °C. Incubation was carried out in a thermoblock at 40 °C for one hour. The amount of MUF and MCA released was then measured using a Hitachi F-2500 spectrophotometer (Tokyo, Japan). For the substrate containing the MUF molecule, the fluorimeter was set at an excitation (EX) wavelength of 318 and an emission (EM) wavelength of 445 nm. For the substrate MCA, a fluorimeter was set up at an emission wavelength of 345 nm and an excitation wavelength of 425 nm. The amount of µmol of MUF/MCA released per hour was taken as a measure of enzyme activity.

### 2.3. Preparation of Chitosan Films

Chitosan (CTS, Mw = 375 kDa, DD = 76%) was dissolved in 0.1 M acetic acid at a 2% concentration. Ellagic acid (EA, M = 302.20 g/mol, anhydrous) and cinnamic acid (CA, M = 162.19 g/mol) were dissolved at a 4% concentration in 0.0015 M NaOH and in ethanol, respectively. Chitosan was mixed with ellagic acid and cinnamic acid solutions separately, and each with two separate weight ratios of dry substances, as follows: 99.8CTS:0.2%EA and 99.6CTS:0.4%EA for ellagic acid, and 99.8CTS:0.2%CA and 99.6CTS:0.4%CA for cinnamic acid. Solutions (40 mL) were mixed on a magnetic stirrer for 1 h and placed on a plastic holder (10 cm × 10 cm). Thin films with 0.050 ± 0.005 mm of thickness were obtained by solvent evaporation. Chitosan-based film without modifications was obtained and studied as a control. Chitosan films were prepared in the Department of Biomaterials and Cosmetics Chemistry, Faculty of Chemistry, Nicolaus Copernicus University in Torun.

#### 2.3.1. Moisture Content

The moisture content of the prepared films was determined using moisture analyzer Radwag MA 50.X2.IC.A (Radom, Poland). Samples (4 cm × 4 cm) were placed on a pan and weighed, followed by a drying process for 10 min at 105 °C. After 10 min of drying, the samples were weighed again. Changes in the samples’ weights were determined as moisture content. The results are expressed as grams of water per 100 g of a dry sample. Three samples were measured for each kind of film.

#### 2.3.2. Biodegradation of Chitosan Films

Biodegradation of modified chitosan by isolates (T.01, T.05, T.07, T.11 and T.14) was determined by oxygen consumption in the presence of films using OxiTop (WTW, Hamburg, Germany). One hundred ml of sterile medium containing (g/L) sucrose 5, yeast extract 0.5 and 10 fragments (2 cm × 2 cm, total mass 0.5 g) of the chitosan films were placed into a bottle. The medium was inoculated with 0.1 mL fungal suspension (spores concentration 10^5^/mL). Incubation was carried out at 26 °C for 7 days. The control sample contained a polymer-free medium inoculated with the same amount of fungal suspension. During the degradation of the modified chitosan films, the fungi consumed oxygen by releasing CO_2_, which was absorbed by NaOH. Biological activity was expressed as mg of O_2_/L of culture.

### 2.4. Determination of BOD in Soil Containing Modified Chitosan Films after Application of Fungi

Biological oxygen demand (BOD) is a measure of the biodegradation of polymers in the natural environment [27]. The biodegradation of the chitosan films in the soil after the application of *Trichoderma* strains was determined using the OxiTop system [16], according to the modified procedure described previously. One hundred grams of soil was transferred to an OxiTop system jar, chitosan film fragments (2 cm × 2 cm) were added and inoculated with fungal spores (1 mL spores, 10^5^ CFU/mL). Incubation was carried out at 26 °C for 21 days. The study was carried out in the following variants: variant 1—biodegradation of chitosan films by native soil microorganisms, variant 2—biodegradation of chitosan film after application of isolate T.07, variant 3—biodegradation of chitosan film after application of isolate T.14, and variant 4—biodegradation of chitosan film after application of a consortium composed of T.07 and T.14 fungi. The control sample contained soil without chitosan films and without supplementation with fungal culture. The biodegradation of chitosan films was determined by oxygen consumption and expressed as mgO_2_/kg of fresh soil. The physicochemical parameters of the soil are presented in Table 1. The analysis was performed by the District Chemical and Agricultural Station in Bydgoszcz.

### 2.5. Determination of Enzyme Activity after Application of Fungi to Soil

Soil without chitosan films (control), soil containing chitosan films without fungal supplementation, and soil with chitosan films with fungal supplementation were used for the study. Enzymatic activity (aminopeptidase, lipase, chitinase, α- and β-glucosidase) was determined as described above (Section 2.2). Previously, 10 g of soil had been diluted ten times with saline (0.9% NaCl) and shaken for 20 min.

### 2.6. Statistical Analysis

All analyses were carried out in three replicates. Statistical analysis was conducted using Past v. 3.08 [28]. The Kruskal–Wallis test and the Mann–Whitney paired test were used.

## 3. Results

### 3.1. Moisture Content

The moisture content of the tested films is presented in Table 2, revealing a significant increase upon the addition of ellagic acid and cinnamic acid. Interestingly, the higher the addition of ellagic acid, the lower the water content observed in the film. Conversely, an opposite trend was noted for cinnamic acid, where higher additions led to increased moisture content. This phenomenon is attributed to the distinct hydrophilicity of the studied phenolic acids. The parameter assumes significance in packaging applications, necessitating a delicate balance in order to maintain homeostasis with the packaged food and to prevent undesirable moisture transfer [29].

### 3.2. Enzyme Activity of Isolates from the Soil of the Kampinos Forest

The fungi isolated from the soil had different activities (Table 3). All isolates were most active lipolytically and chitinolytically. Lipase activities ranged from 36.19 U/h to 62.21 U/h. Isolates T.07 and T.14 showed the highest lipolytic activity (55.78 U/h and 62.21 U/h, respectively). In contrast, the chitinase activity of the isolates ranged from 17.27–43.03 U/h. With the highest activity observed for T.07 and T.14 (43.03 U/h and 41.27 U/h, respectively). All tested isolates had relatively low α and β-glucosidase activity.

### 3.3. Respirometric Activity of Fungi (mgO_2_/L) in the Presence of Modified Chitosan Films

Among the fungal isolates tested, only two strains used chitosan films as a source of energy: isolates T.07 and T.14 (Table 4). The respirometric activity of these isolates in the presence of chitosan film and modified chitosan film was similar. In the presence of pure chitosan, the oxygen consumption of T.07 was 106 mgO_2_/L and that of T.14 was 90 mgO_2_/L. The introduction of cinnamic acid and ellagic acid into the chitosan film significantly reduced the respirometric activity of both isolates. Oxygen consumption by the fungi was lowest in the medium consisting of chitosan containing 0.4% cinnamic acid (16–18 mgO_2_/L). Chitosan film with the addition of ellagic acid was also poorly consumed by the isolates and was better than chitosan film with cinnamic acid.

### 3.4. Biodegradation of Modified Chitosan Film in Soil after Fungal Application

**Figure 2:**
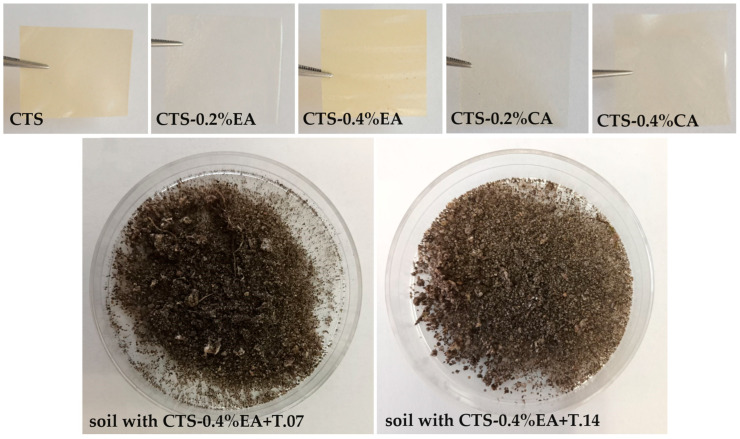
The chitosan films before biodegradation and soil samples with broken films after fungal application.

Biodegradation of the modified chitosan film after 21 days in the soil was determined by oxygen consumption (Table 5). When analyzing the oxygen consumption values of the microorganisms in the presence of the tested chitosan materials, it was found that the soil microorganisms without the application of fungal isolates were respirometrically active. The addition of fungi to the soil increased oxygen consumption. At the same time, significant differences in oxygen consumption were observed between the fungi introduced and the type of film. The respirometric activity in soil containing pure chitosan film before and after fungal application was similar. The modified chitosan film significantly influenced the oxygen consumption of microorganisms in the soil. Chitosan containing 0.4% cinnamic acid significantly inhibited respirometric activity. Although application of the tested fungi to the soil increased oxygen consumption in the presence of CTS-0.4%CA, oxygen consumption was lower compared with pure chitosan film. In contrast, the introduction of ellagic acid into the chitosan film also reduced oxygen uptake by soil microorganisms, but to a lesser extent. The modified chitosan film had completely broken down after 21 days in the soil, making it impossible to perform physicochemical analysis (Figure 2).

### 3.5. Changes in Enzyme Activity in Soil after Fungal Application

The introduction of fungi into soil containing modified chitosan film variably altered the enzymatic activity of the soil (Table 6). No statistically significant differences in glucosidase activity were observed after the introduction of chitosan films and fungi into the soil. The activity of these enzymes was similar and was the lowest compared with other hydrolytic enzymes. Aminopeptidase activity in the soil was also similar. Only the soil containing CTS-CA before and after fungal application showed a decrease in the activity of these enzymes. Chitinase activity in the soil varied the most. In soil without chitosan films and fungi, chitinase activity was 9.3 U/h. In soil after the introduction of chitosan films with ellagic acid and cinnamic acid without fungal application, significantly lower chitinase activity was found than in soil after fungal application. Even the higher highest concentration of EA and CA added to the chitosan film (0.4%) did not inhibit enzyme activity.

### 3.6. Identification of Fungi

Based on the amplification of the ITS regions, isolates from Kampinoski National Park soil were classified in the genus *Trichoderma* (Table 7). The resulting sequences designated T.01, T.05, T.07 I T.14 have been deposited with GeneBank NCBI under the following accession numbers: T.01—PP179221, T.05—PP179220, T.07—PP179233, T.11—PP179212 and T.14—PP179234.

## 4. Discussion

Chitosan has potential applications across various industries, such as agriculture, as well as in the nutraceutical and pharmaceutical sectors. Chitosan films and coatings have been extensively studied due to their biodegradable, antimicrobial, and non-toxic nature, and their origin from renewable sources [30,31]. These films can serve as active packaging sheets or as nanoparticle composites, with several techniques employed to alter chitosan to enhance its food-packaging properties [32,33]. The incorporation of various additives, such as essential oils, phenolic acids, and plant extracts, into chitosan has been found to significantly enhance its antibacterial and antioxidant properties. This emerging practice of utilizing coating to extend the shelf life of products is currently gaining momentum [34,35,36,37]. In our study, ellagic acid and cinnamic acid were introduced into the chitosan film at concentrations of 0.2 and 0.4%. Ellagic acid is well known for its antioxidant properties and potential health benefits, making it a valuable addition to bioactive packaging materials aimed at improving the functionality and preservation of packaged food products [38]. Cinnamic acid also holds promise for applications in packaging materials. It, along with its derivatives, has been studied for its antimicrobial, antioxidant, and anti-fungal properties [21,39,40]. Chitosan active sheets and nanoparticle composites are also utilized as plastic mulch to enhance soil quality, and as seed coatings during storage, providing additional functionality beyond passive barriers, such as protection against microbial growth, oxidation, and other forms of deterioration [32,33,41,42]. While they inhibit the growth of certain pathogens, they must also be biodegradable to avoid environmental pollution. The main challenge in polymer biodegradation lies in the presence of biocidal compounds, which not only hinder the growth of potential foodborne microorganisms but also affect environmental microorganisms upon disposal. Therefore, it is important to develop strategies that facilitate effective biodegradation. Microorganisms capable of synthesizing enzymes to degrade the components of packaging materials appear to offer the most promising approach. In our study, we selected fungi from the genus *Trichoderma*, which display high enzymatic activity. Our research has shown that these fungi synthesize key enzymes involved in the biodegradation of organic matter, with chitinases exhibiting the highest activity. The enzyme complex produced by the *Trichoderma* fungi consists of chitinases, β-glucanases, cellulases, and proteases, which are involved in hydrolysis [43,44]. According to Abdenaceur et al. [45], the *Trichoderma* species exhibits varying levels of enzymatic activity, producing amylases, pectinases, lipases, and phosphatases. The role of these fungi has previously been studied [46]. Fungi of the genus *Trichoderma* have been studied in biocontrol, but in our research we propose a more extensive use of these fungi. Studies have shown that they produce chitosanases that are involved in the degradation of chitosan [47]. Our study has shown that only two of the fungi tested degraded the modified chitosan films. Our previous studies have also shown that not all fungi have the ability to degrade modified chitosan [16]. *Trichoderma atroviride* TN1 and *Trichoderma citrinoviride* TN3 best metabolized the chitosan films containing tannic acid, ferulic acid, and gallic acid. The contribution of fungi to the degradation of chitosan films remains relatively understudied. *Trichoderma* species produce different types of chitinolytic enzymes and chitosanases. It would seem that it is not only chitosanases that are able to hydrolyze chitosan. According to Beer et al. [48], chitosan undergoes significant hydrolysis by cellobiose hydrolases, leading to the formation of water-soluble oligomers with a molecular weight ranging from 10 to 15 kDa. The production of cellulases, chitinases, glucanases, xylanases, and protease enzymes by *Trichoderma* spp. has garnered considerable attention due to their remarkable capabilities in this regard [49]. This study has shown that *Trichoderma* isolates with the highest chitinase activity also most efficiently decomposed modified chitosan films. Studies on the enzymatic activity of *Trichoderma,* as well as on the decomposition of chitosan films, have selected isolates for introduction into the soil to improve the biodegradation of chitosan materials. Our study has shown that the introduction of single isolates of *Trichoderma* sp. T.07 and *Trichoderma koningii* T.14, or their consortia, improved the biodegradation of the modified chitosan film. Chitosan films modified with ellagic and cinnamic acid disintegrated into small fragments in the soil after 21 days in OxiTop. However, this does not indicate complete decomposition to CO_2_, but to microparticles which are then further broken down in the soil. In this form, they can be more readily utilized by microorganisms. We do not exclude the possibility that, through the breakdown of the chitosan film, CA and EA are released in the soil. These compounds can be metabolized by *Trichoderma*. Chen et al. [50] have reported that, after 45 days, CA in the rhizospheres of continuously cropped cucumbers was 100% degraded. In their study, the authors used the bioaugmentation of *Trichoderma harzianum* SQR-T037. Ellagic acid is a product of the degradation of ellagitannins with the enzymes known as ellagitannases, which are synthesized by *Aspergillus* fungi [51]. This research may indicate that these fungi use EA as a source of energy.

The introduction of biocidal polymers into the soil can also impact enzymatic activity. Our studies have revealed that modified chitosan films do not affect the activity of important hydrolytic enzymes. Enzymatic activities in the soil are regarded as biological indicators, especially in polluted soil. Cinnamic acid and ellagic acid have antimicrobial properties and can inhibit the growth of enzyme-producing soil microorganisms. According to He et al. [52], cinnamic acid effectively inhibits the growth of *Trichoderma harzianum*. Stanek et al. [53] have reported that changes in soil microbial characteristics and enzymatic function could potentially be linked to significant quantities of ellagic acid or ferulic acid. Certain phenolic compounds, such as quercetin and chlorogenic acid, found in the soil, have been found to show a positive correlation with soil microbial parameters and/or enzymatic activity. This correlation implies that these compounds may possess either antimicrobial or stimulatory properties. In our study, the application of modified chitosan films had the significant effect of increasing chitinase activity. Fungi of the genus *Trichoderma* are known for synthesizing chitinolytic enzymes, and it is likely that the tested materials did not inhibit the growth of these fungi in the soil.

The selection of fungi to increase biodegradation efficiency is a first step towards further research into formulation development. The activity of fungi in the environment is influenced by a multitude of biotic and abiotic factors. Further research should focus on developing a carrier that allows the maintenance of high activity and viability of fungi. Microencapsulation has the potential to maximize efficacy. Maruyama et al. [54] have reported that encapsulation of *T. harzianum* led to improvement in the chitinolytic and cellulosic activity. According to the authors, calcium alginate microparticles could be effective in protecting *T. harzianum* against ultraviolet radiation and presumably other abiotic factors in the environment.

## 5. Conclusions

Antimicrobial agents can be added to natural biopolymer packaging to further improve it and to create active packaging. The natural origin and functionality of ellagic and cinnamic acid make them appealing components for biodegradable food packaging. Potential bioactive agents can be used in biodegradable packaging as a promising solution to food spoilage problems, providing an environmentally friendly alternative to traditional packaging. At the same time, biodegradable packaging is safer for the environment into which it is disposed of after use. Selected fungi belonging to the genus *Trichoderma,* designated as T.07 and T.14, can increase the biodegradation efficiency of mseodified chitosan films in soil. Further research into the development of a carrier to preserve the activity of *Trichoderma* fungi may offer more opportunities for their use as a bioinoculant.

## Figures and Tables

**Table 1:** Physicochemical properties of the soil.

Parameters	Values
pH in KCl	7.1
C-org (%)	1.18
N-min (kg/ha)	345
N-NH_4_ (mg/kg)	2.76
N-NO_3_ (mg/kg)	29.03
P_2_O_5_ (mg/100 g)	29
Mg (mg/100 g)	6.6
K_2_O (mg/100 g)	28

**Table 2:** The moisture content of chitosan-based films with ellagic acid and cinnamic acid addition (* significantly different from CTS, *p* < 0.05).

Chitosan Films	Moisture Content (g/100 g)
CTS	12.95 ± 1.61
CTS-0.2%CA	13.63 ± 2.19 *
CTS-0.4%CA	16.24 ± 1.31 *
CTS-0.2%EA	15.97 ± 3.24 *
CTS-0.4%EA	13.17 ± 0.21 *

**Table 3:** Enzyme activity (U/h) of fungi from the soil of the Kampinos Forest.

Isolates	Aminopeptidase	Lipase	Chitinase	α-Glucosidase	β-Glucosidase
T.01	11.58 ± 0.02 ^b^	36.75 ± 0.04 ^b^	19.91 ± 0.02 ^b^	2.91 ± 0.02 ^b^	4.01 ± 0.03 ^a^
T.05	19.95 ± 0.01 ^a^	39.64 ± 0.03 ^b^	17.27 ± 0.03 ^b^	2.98 ± 0.07 ^b^	4.05 ± 0.1 ^a^
T.07	20.15 ± 0.03 ^a^	55.78 ± 0.02 ^a^	43.03 ± 0.02 ^a^	4.18 ± 0.03 ^a^	4.09 ± 0.1 ^a^
T.11	11.75 ± 0.01 ^b^	36.19 ± 0.03 ^b^	18.47 ± 0.02 ^b^	3.78 ± 0.04 ^a^	4.31 ± 0.06 ^a^
T.14	12.45 ± 0.03 ^b^	62.21 ± 0.02 ^a^	41.27 ± 0.02 ^a^	4.11 ± 0.08 ^a^	4.09 ± 0.07 ^a^

Values are expressed as mean ± SD (*n* = 3). Different letters in the column indicate statistically significant differences at *p* < 0.05.

**Table 4:** Respirometric activity of fungi (mgO_2_/L) in the presence of modified chitosan films.

Chitosan Films	Isolates				
	T.01	T.05	T.07	T.11	T.14
CTS	0	0	106 ± 2.1 ^aA^	0	90 ± 2.2 ^bA^
CTS-0.2%CA	0	0	58 ± 1.3 ^aB^	0	35 ± 2.3 ^bB^
CTS-0.4%CA	0	0	18 ± 0.9 ^aC^	0	16 ± 3.1 ^aC^
CTS-0.2%EA	0	0	98 ± 2.1 ^aA^	0	88 ± 3.1 ^aA^
CTS-0.4%EA	0	0	47 ± 1.1 ^aB^	0	34 ± 2.1 ^aB^

Values are expressed as mean ± SD (*n* = 3). Different lowercase letters indicate statistically significant differences with *p* < 0.05 between microorganisms. A different uppercase letter indicates a significant difference at *p* < 0.05 between chitosan films. CTS—chitosan, CTS-CA—chitosan modified with cinnamic acid, CTS-EA—chitosan modified with ellagic acid.

**Table 5:** BOD_21_ (mgO_2_/kg) in soil containing modified chitosan films after application of fungi.

Chitosan Films	Variants			
	Native Soil Microorganisms	Isolate T.07	Isolate T.14	Consortium T.07 + T.14
CTS	2189 ± 13.2 ^aA^	2884 ± 35.5 ^aA^	2675 ± 33.3 ^aA^	2929 ± 44.2 ^aA^
CTS-0.2%CA	1008 ± 23.3 ^bB^	1540 ± 33.1 ^aB^	1710 ± 14.2 ^aB^	1765 ± 23.3 ^aA^
CTS-0.4%CA	484 ± 12.9 ^cC^	611 ± 13.9 ^bD^	662 ± 12.8 ^bC^	917 ± 12.1 ^aC^
CTS-0.2%EA	1009 ± 24.5 ^bB^	1926 ± 14.4 ^aC^	1910 ± 13.1 ^aB^	1985 ± 22.9 ^aB^
CTS-0.4%EA	637 ± 14.1 ^bC^	1350 ± 13.3 ^aC^	1321 ± 14.5 ^aB^	1410 ± 12.2 ^aB^

Values are expressed as mean ± SD (*n* = 3). Different lowercase letters indicate statistically significant differences with *p* < 0.05 between microorganisms. A different uppercase letter indicates a significant difference at *p* < 0.05 between chitosan films. CTS—chitosan, CTS-CA—chitosan modified with cinnamic acid, CTS-EA—chitosan modified with ellagic acid.

**Table 6:** Effect of modified chitosan films and fungi bioaugmentation on enzymatic activity in soil.

Variants	Enzyme Activity (U/h)				
	Chitinase	Lipase	α-Glucosidase	β-Glucosidase	Aminopeptidase
soil	9.3 ± 0.13 ^c^	19.32 ± 0.14 ^a^	4.7 ± 0.11 ^a^	3.2 ± 0.11 ^a^	9.3 ± 0.16 ^a^
CTS+soil	16.2 ± 0.13 ^b^	21.5 ± 0.27 ^a^	4.3 ± 0.10 ^a^	3.5 ± 0.14 ^a^	10.1 ± 0.13 ^a^
CTS+soil+T.07	27.3 ± 0.11 ^a^	11.7 ± 0.10 ^c^	4.6 ± 0.12 ^a^	3.8 ± 0.13 ^a^	9.8 ± 0.16 ^a^
CTS+soil+T.14	25.5 ± 0.60 ^a^	22.2 ± 0.02 ^a^	5.5 ± 0.17 ^a^	2.0 ± 0.16 ^b^	10.1 ± 0.13 ^a^
CTS+soil+consortium	26.1 ± 0.23 ^a^	26.9 ± 0.03 ^a^	4.6 ± 0.41 ^a^	4.1 ± 0.13 ^a^	10.6 ± 0.03 ^a^
CTS-0.2%CA+soil	8.3 ± 0.11 ^c^	22.9 ± 0.03 ^a^	4.9 ± 0.13 ^a^	4.2 ± 0.13 ^a^	10.1 ± 0.13 ^a^
CTS-0.2%CA+soil+T.07	24.3 ± 0.12 ^a^	17.8 ± 0.11 ^b^	5.3 ± 0.11 ^a^	4.8 ± 0.12 ^a^	10.1 ± 0.13 ^a^
CTS-0.2%CA+soil+T.14	26.6 ± 0.17 ^a^	17.3 ± 0.06 ^b^	4.2 ± 0.11 ^a^	4.8 ± 0.12 ^a^	9.0 ± 0.14 ^a^
CTS-0.2%CA+soil+consortium	20.6 ± 0.22 ^b^	16.4 ± 0.11 ^b^	4.0 ± 0.14 ^a^	4.9 ± 0.11 ^a^	10.9 ± 0.11 ^a^
CTS-0.4%C+soil	8.9 ± 0.28 ^c^	19.9 ± 0.08 ^a^	4.7 ± 0.11 ^a^	4.2 ± 0.18 ^a^	6.7 ± 0.12 ^b^
CTS-0.4%CA+soil+T.07	15.2 ± 0.11 ^b^	14.0 ± 0.04 ^b^	4.8 ± 0.11 ^a^	4.4 ± 0.11 ^a^	7.4 ± 0.13 ^b^
CTS-0.4%CA+soil+T.14	18.3 ± 0.21 ^b^	23.3 ± 0.01 ^a^	4.6 ± 0.13 ^a^	4.9 ± 0.11 ^a^	6.5 ± 0.13 ^b^
CTS-0.4%CA+soil+consortium	18.5 ± 0.57 ^b^	25.1 ± 0.01 ^a^	4.3 ± 0.12 ^a^	4.6 ± 0.11 ^a^	6.1 ± 0.16 ^b^
CTS-0.2%EA+soil	9.8 ± 0.21 ^c^	21.3 ± 0.11 ^a^	4.8 ± 0.11 ^a^	5.1 ± 0.11 ^a^	9.7 ± 0.11 ^a^
CTS-0.2%EA+soil+T.07	21.0 ± 0.22 ^a^	17.3 ± 0.10 ^b^	4.5 ± 0.20 ^a^	4.9 ± 0.10 ^a^	9.0 ± 0.12 ^a^
CTS-0.2%EA+soil+T.14	20.1 ± 0.10 ^a^	20.0 ± 0.11 ^a^	4.4 ± 0.20 ^a^	5.0 ± 0.11 ^a^	8.1 ± 0.10 ^a^
CTS-0.2%EA+soil+consortium	19.5 ± 0.10 ^a^	17.6 ± 0.21 ^b^	4.2 ± 0.10 ^a^	4.6 ± 0.21 ^a^	10.2 ± 0.21 ^a^
CTS-0.4%EA+soil	8.2 ± 0.11 ^c^	16.8 ± 0.10 ^b^	4.1 ± 0.11 ^a^	3.0 ± 0.11 ^a^	4.3 ± 0.23 ^c^
CTS-0.4%EA+soil+T.07	16.2 ± 0.10 ^b^	16.7 ± 0.20 ^b^	4.0 ± 0.12 ^a^	3.8 ± 0.10 ^a^	5.8 ± 0.21 ^b^
CTS-0.4%EA+soil+T.14	17.3 ± 0.21 ^b^	17.5 ± 0.20 ^b^	4.1 ± 0.10 ^a^	3.3 ± 0.20 ^a^	6.3 ± 0.11 ^b^
CTS-0.4%EA+soil+consortium	16.6 ± 0.10 ^b^	16.0 ± 0.10 ^b^	4.0 ± 0.21 ^a^	3.7 ± 0.20 ^a^	5.5 ± 0.21 ^b^

Values are expressed as mean ± SD (*n* = 3). Different letters in the column indicate statistically significant differences at *p* < 0.05. CTS—chitosan, CTS+AC—chitosan modified with cinnamic acid, CTS+AE—chitosan modified with ellagic acid.

**Table 7:** Identification of fungal isolates.

Symbols	Top-Hit Taxon	Top-Hit Strain	Similarity (%)	Completeness (%)
T.01	*Trichoderma atroviride*	II ODh	97.55	97
T.05	*Trichoderma atroviride*	MA3634	88.12	80
T.07	*Trichoderma* sp.	SDAS203127	94.29	96
T.11	*Trichoderma hamatum*	NECC30476	92.61	87
T.14	*Trichoderma koningii*	S54	94.32	98

## Data Availability

The data presented in this study are available on request from the corresponding author. The data are not publicly available due to privacy restrictions.
